# Nuclear and nucleolar activity of linker histone variant H1.0

**DOI:** 10.1186/s11658-016-0014-0

**Published:** 2016-08-24

**Authors:** Andrzej Kowalski

**Affiliations:** Department of Biochemistry and Genetics, Institute of Biology, Jan Kochanowski University, Świętokrzyska 15, 25-406 Kielce, Poland

**Keywords:** Chromatin, Histone H1.0, Histone H1 subtypes, Intrinsic protein disorder, Nucleus, Nucleolus, Protein-protein interaction

## Abstract

Histone H1.0 belongs to the class of linker histones (H1), although it is substantially distinct from other histone H1 family members. The differences can be observed in the chromosomal location and organization of the histone H1.0 encoding gene, as well as in the length and composition of its amino acid chain. Whereas somatic (H1.1–H1.5) histone H1 variants are synthesized in the cell cycle S-phase, histone H1.0 is synthesized throughout the cell cycle. By replacing somatic H1 variants during cell maturation, histone H1.0 is gradually deposited in low dividing cells and achieves the highest level of expression in the terminally differentiated cells. Compared to other differentiation-specific H1 histone (H5) characteristic for unique tissue and organisms, the distribution of histone H1.0 remains non-specific. Classic investigations emphasize that histone H1.0 is engaged in the organization of nuclear chromatin accounting for formation and maintenance of its nucleosomal and higher-order structure, and thus influences gene expression. However, the recent data confirmed histone H1.0 peculiar localization in the nucleolus and unexpectedly revealed its potential for regulation of nucleolar, RNA-dependent, activity via interaction with other proteins. According to such findings, histone H1.0 participates in the formation of gene-coded information through its control at both transcriptional and translational levels. In order to reappraise the biological significance of histone H1.0, both aspects of its activity are presented in this review.

## Background

The majority of histone H1 subtypes, including those that are specific of sperm (H1t, H1T2, and H1LS1) or oocytes (H1oo) as well as those active in the somatic cells (H1.1–H1.5 and H1X), can be characterized as having more than 200 amino acids in chain [1], with the largest share of the Ala and Lys residues. For example, the molecules of mammalian somatic histone H1 subtypes (H1.1–H1.5) comprise of average 219 amino acid residues [1], in which about 20.7 and 25.18 % correspond to residues of Ala and Lys, respectively [2]. The histone H1.0 molecule is generally shorter than the remaining H1 variants. Although the 194 amino acids long mammalian histone H1.0 contains approximately one-third fewer Ala residues, it also possesses about a half larger number of Ser residues, as well as Arg, Phe, Ile and Leu residues, which are virtually absent in the remaining histone H1 somatic subtypes (Table 1) [2, 3]. Consequently, the shortest histone H1.0 molecule, possessing diverged C-terminal domain with a high proportion of basic amino acid residues, exhibits different chromatin binding properties compared to most other histone H1 variants [4]. Also, the histone H1.0 discontinuous subdomains were found to affect both DNA binding and chromatin folding [5]. While genes encoding human somatic H1 histones are usually clustered on the short arm of chromosome 6, and their non-polyadenylated mRNAs contains hairpin loop terminal element, the histone H1.0 encoding gene is separately located on the long arm of chromosome 22, and its polyadenylated mRNA comprises an extended 3′ non-coding segment [6]. Since synthesis of ubiquitous H1 histones is coupled to the S-phase, they are often termed replication-dependent histone H1 variants [1]. In contrast to the H1 histones, which are active during embryonal stages, and to those which appear consecutively in somatic cells, histone H1.0 is accumulated along with cell maturation and achieves maximal expression in the non-proliferating cells [7]. Therefore, histone H1.0 is known as differentiation-specific histone H1 variant, which may be found in many vertebrates, mainly in mammals [8, 9] and amphibians [10], but also in fish [11] and reptiles [12].

**Table 1:** The overview of intrinsic disorder features attributable to the mouse histone H1.0

Physic-chemical and structural features	Attributes of intrinsic disorder
Amino acid composition^a^	Enriched aa: Ala (14.9 %), Lys (28.9 %)
	Depleted aa: Asp (2.1 %), Glu (2.6 %), Phe (1.1 %)
Grand average hydrophobicity^b^	Negative (score −1.073)
Disorder degree^c^	Ratio – 0.62; disordered aa – 120/whole aa – 194
Domain disorder level^c^	N- and C- – 100 %, Globular – 0 %
Long disordered regions^d^	Score – 0.8153, threshold score – 0.5380
MoRFs content^e^	Three elements (6, 9 and 10 aa)
ANCHOR binding regions^f^	Seven elements (12, 11, 14, 9, 18, 6 and 55 aa)
Low-complexity regions^g^	18 aa N-/86 aa C-

*aa* amino acids, *N* (N-terminal), *C* (C-terminal), *Globular* histone H1.0 domains

^a^Composition Profiler (Vacic et al. [63])

^b^ExPasy proteomic server (ProtParam, Gravy tool)

^c^PrDOS (Ishida and Kinoshita [64])

^d^SLIDER (Peng et al. [65])

^e^MoRFpred (Disfani et al. [66])

^f^ANCHOR (Dosztànyi et al. [54])

^g^SEG server (Wootton [67])

The importance of histone H1.0 in determining the activity of nuclear chromatin has been confirmed at both nucleosomal [13] and higher order organization level [5]. In contrast to other H1 histones, H1t [14] and H1c [15], histone H1.0 strongly inhibits DNA replication [14] and reduces the RNA transcript levels [15]. Histone H1.0 functions are attributable to its globular domain [16], as well as to the flanking domains [13]. One of them, the C-terminal domain, is intrinsically disordered [2, 3] and, therefore, capable of multiple interactions [17] with DNA and partnering proteins. The flexible C-terminal domain binding to DNA and/or partnering proteins is coupled to its structural change repeatedly affected by post-translational modifications. The histone H1.0 C-terminal domain is modified mainly by phosphorylation. In dependence on the amount of modified amino acid residues, phosphorylation contributes to the formation of condensed and/or relaxed chromatin conformation [18]. Even though the previous findings confirmed both a high concentration of histone H1.0 in the dense chromatin areas surrounding the nucleoli [19] and its localization in perinucleolar regions [20], the possible nucleolar activity of histone H1.0 remained unexplained. However, the latest experiments revealed a peculiar set of histone H1.0 interactome, in which over a hundred candidate H1.0-binding proteins engaged in the RNA metabolism were identified in the nucleolus [21]. The presence of histone H1.0 in nucleolar chromatin was also confirmed by its enriched deposition at the NADs and at the NORs [22]. In accordance with the above evidence, the histone H1.0 is responsible for both protein-DNA and protein-protein interactions in nucleus and nucleolus, respectively. Thus, this peculiar histone H1 subtype might be perceived as one of the main chromatin modulators affecting transcriptional and translational regulation of gene expression.

### Histone H1 class

Histone H1, a key protein engaged in the organization and stabilization of chromatin structure [1, 23], is composed of several variants (subtypes) existing in a different number in various organisms. Usually, the histone H1 set ranges from four to seven subtypes in animals and plants, reaching a maximum of eleven proteins in mammals [24]. The mammalian histone H1 subtypes are categorized according to its expression in the somatic (H1.1–H1.5 and H1x) and germ (H1t, H1T2, H1oo) cells, of which subtypes H1.1–H1.5 and H1t are synthetized in the replication-dependent mode while synthesis of the subtype H1.0, H1x, H1T2 and H1oo is not linked to the replication [1, 25]. The histone H1 variants play a common role in the nucleosome orientation and in the creation of compacted, transcriptionally inert, higher order chromatin structure. Individually, H1 subtypes affect local chromatin organization and function, contributing to the gene activation or repression [24]. Therefore, both redundancy and specificity is attributable to the histone H1 functioning [25].

A redundant effect was shown due to the compensation of eliminated H1.0 subtype by the remaining set of H1 variants (H1c, H1d and H1e) in the H1.0-knock out mice strains [26]. The compensation also occurs in the doubly H1-deficient mice strains, simultaneously deprived of the subtypes H1.0/H1.c, H1.0/H1.d and H1.0/H1e [27]. Whereas no phenotypic abnormality of mutated animals was observed, the compensation corresponding to the up-regulation of histone H1 variants is needed to maintain the normal stoichiometry between histone H1 and nucleosome. A reduction of total histone H1 content resulting in the decrease of nucleosome repeat length and in the formation of less compacted, more variable chromatin conformation were observed in triple-H1 (H1.c, H1.d and H1.e) null mice embryonic cells. Elimination of three histone H1 variants leading to the embryonic lethality indicate a limitation of histone H1 subtypes redundancy and a tendency to maintenance of the correct histone H1 content appropriate for regular development [28]. However, no compensatory effect was noted after inducible depletion of the single H1 subtypes in the breast cancer cells. The observed subtype-specific dependent changes in the nucleosome spacing and chromatin organization as well as in the repressed expression of cell cycle genes and cell proliferation defects suggest that histone H1 subtypes might be functionally differentiated [29].

Many studies support the theory that histone H1 variants have distinct role in chromatin, triggering the individual impact on some of cellular processes (for recent review, see [30–32]). Specialization of histone H1 variants might be inferred from their individual characteristics related to the strength of binding DNA and chromatin. Similarly, a difference in the degree of sequence conservation and distinct genomic location may be linked to the varied roles of histone H1 subtypes in chromatin regions or cell types. FRAP experiments have proved that the mammalian histone H1 subtypes bind chromatin with different affinity with respect to their mobility, influenced by the lengths of C-terminal domain [33]. Histones H1.1 and H1.2 having the shortest C-terminal domain, rapidly bind and dissociate between chromatin sites in contrast to histones H1.3, H1.4 and H1.5, which have a longer C-terminal domain and exchange more slowly. Thus, histone H1 subtypes differ in their chromatin binding properties, representing low (H1.1 and H1,2), moderate (H1.3) and high (H1.4 and H1.5) binding affinity. Interestingly, histone H1.0 possessing the shortest C-terminal domain, has moderate binding affinity, due to a higher content of the Lys residues and a high density of the DNA-binding motifs. A distinct ability of histone H1 subtypes to stabilize chromatin folding was confirmed by Clausell and colleague [34], who monitored H1 histones assembled with pre-blastodermic *Drosophila melanogaster* embryo extracts. Using AFM they found that chromatin compaction is strongly influenced by the subtype H1.0, H1.4, H1.5 and H1x. A relaxed chromatin structure is maintain by the subtypes H1.1 and H1.2 and to a lesser extent by the subtype H1.3. This suggest that operation of distinct H1 subtypes is required for a specific chromatin activity. Both chromatin distribution and genomic localization seems to reflect the individual function of histone H1 subtypes. They were detected as differently distributed in the chromatin regions, localized both in euchromatin (H1.1–H1.3) and in the heterochromatin (H1.4, H1.5) area. The histone H1.0 associated mainly with euchromatin was also found as enriched in a single heterochromatin domain [33]. Recently, Izzo et al. [35] employed DamID profiling to map histone H1 genomic distribution in the IMR90 cells. The histone H1 subtypes DamID binding profiles were distinct around the domains enriched in the epigenetic marks and chromatin regulators, designating active (H3K36me3 and H3K4me3) and repressed (PcG domains enriched in H3K27me3 and HP1 domains enriched in H3K9me3) chromatin regions. Whereas histone H1.2–H1.5 were depleted from active domains and enriched in HP1, the histone H1.1 more abundant in active domains was preferentially located in PcG. Thus, histone H1.1 may play a special role forming both a more condensed (repressed) and less compacted (active) chromatin state [35]. The histone H1.1 functional differentiation might be also due to its larger divergence, compared to the more conserved histone H1 somatic subtypes [36]. This functional uniqueness concerns not only histone H1.1. An interesting example is the histone H1.2 which, unlike the other histone H1 subtypes, is mainly associated with gene poor regions and chromosomes in the breast cancer cells [37]. Nevertheless, it might maintain both transcriptionally active and inactive chromatin. Whereas transcriptional activation promoted by H1.2 subtype refers to the cooperation with Cul4A E3 ubiquitin ligase and PAF1 elongation complexes [38], the inactivation of transcription is due to the recognition of methylated histone H3 (H3K27me3), mediated by the EZH2 subunit of PRC complex, to form compacted chromatin and gene silencing [39].

Although a partial redundancy is linked to the histone H1 function [25], a large number of recent studies indicate the individual impact of histone H1 subtypes on DNA binding and chromatin compaction related to the regulation of gene expression [1, 24, 30, 32]. In contrast to the findings regarding histone H1 subtypes activity in the nucleus, studies focusing on histone H1 nucleolar activity represent a giant step in the exact determination of its functioning. In this field, attention should be paid on the histone H1.0, the first histone H1 subtype recognized as associated with the organization and function of the nucleolus.

### Histone H1.0 in nuclear chromatin

The initial identification of histone H1.0 in non-dividing cells [40] and subsequent observations of its accumulation, inversely correlated with the rate of cell division [41, 42], led to the conclusion that histone H1.0 is essential in the formation and stabilization of condensed higher order chromatin structure in the terminally differentiated cells. The occurrence of histone H1.0 in reconstituted chromatin restricts the action of micrococcal nuclease, allowing for conformational changes that enable formation of solenoidal and, consequently, more compacted chromatin structure [43]. However, another reconstitution experiment proved that the transcriptionally active chromatin fraction is almost twofold enriched in histone H1.0, as compared to the one containing untranscribed sequences [44]. The apparent contradiction of such results explains analyzing different chromatin subfractions, corresponding to the inactive β-fetoprotein gene [43] and to the active gene of albumin [44]. This might indicate that histone H1.0 can specifically influence the individual gene expression. Such a notion was confirmed in the experiments performed by Brown and colleagues, who compared the variant-specific activity of histone H1.0 and histone H1c [14, 45] in the series of transformants overproducing combinations of H1 variants domain-shift hybrids. The RNase protection assays revealed a significant reduction of transcript levels in the transformants overproducing histone H1.0. It consequently led to limited expression of both immediate early serum response genes (c-*fos* and c-*myc*) [45] and the constitutive genes (cyclophilin, β-actin and GAPDH) [14]. Reduced gene activity can be triggered by a more compacted chromatin state induced through the overproduction of histone H1.0 [15, 46]. The histone H1.0-dependent gene expression was also confirmed by microarray data [29], showing that the genes might be both up- and down- regulated by histone H1.0. Among the genes uniquely targeted by histone H1.0 are those whose function is associated with amino acid metabolism, protein synthesis and protein trafficking. The modulation of gene expression is dependent on a peculiar chromatin state at a given gene locus, determined by transient removal and/or transient binding of histone H1.0. This leads to the formation of chromatin regions that are accessible and/or inaccessible, respectively, for transcription [29]. Likewise, both activation and repression of genes linked to cell cycle were reported in the histone H1.0 depleted cells. Whereas depletion of H1.0 triggered activation of about 20 genes linked to the cell cycle and led to the repression of 2 genes only, it can be foreseen that histone H1.0 can cause a stronger repressive effects in chromatin [29]. A similar conclusion concerning gene activity controlled by histone H1.0 comes from the studies conducted by Therme et al. [47], in which the H1.0 was detected as a prevalent regulator of genes affecting differentiation and pluripotency in the human ES cells. In agreement with these findings, histone H1.0 affects cell differentiation not only by retarding silencing of the genes related to pluripotency, but also by influencing transcription of the genes associated with differentiation. Whereas potential histone H1.0-dependent transcriptional activation cannot be excluded, the histone H1.0 engagement in the induction of transcriptional repression seems to be much more probable, due to its increasing accumulation at the differentiation-specific genes promoters [47].

**Fig. 1:**
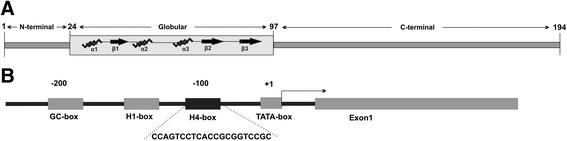
Structural organization of histone H1.0 molecule and its gene promoter. In **a**, the domain structure of the histone H1.0 molecule is presented along with the length of amino acid residues. In the globular domain, α-helical (α1–α3) and β-stranded (β1–β3) elements forming secondary structure are depicted. In **b**, the elements of the gene promoter located within 200 bp (−200) upstream of the transcription start site (+1) are presented. A H4-box sequence unique for histone H1.0 is shown [58]

A disparate affinity for chromatin and, subsequently, the distinct effects of its operation are implemented individually by distinct histone H1.0 domains (Fig. 1). The overproduction of histone H1.0, which results in the gene repression [15], takes place thanks to a peculiar sequence of histone H1.0 globular domain that strengthen the binding of DNA by reinforcing a charge density. However, due to a substantial content of positively charged amino acids, the C-terminal domain of mutated histone H1.0 mediates the formation of a highly compacted chromatin state and the inhibition of DNA replication [14]. These findings together with other data stressing the individual impact of histone H1.0 globular [48] and terminal [13] domain on nucleosome surface interaction and chromatin binding affinity, respectively, indicate that histone H1.0 may evoke a characteristic chromatin environment by differential interactions with both DNA and potential partner proteins. Mutated histone H1.0 analyzed by photobleaching microscopy revealed two clusters of amino acid residues forming DNA-binding sites within the globular domain. Binding site 1 contacts DNA backbone outside the nucleosome dyad while binding site 2 interacts with one DNA strand away from the nucleosomal core DNA. According to this off-dyad binding mode of histone H1.0 globular domain-DNA interaction, the conserved DNA structure at the nucleosome dyad is recognized by structurally stable site 1 residues (Arg74, His25, Lys47, Lys69, Lys73 and Lys85), while diverse linker DNA regions are positioned by the residues (Arg42, Arg94 and Arg97) at site 2 [16]. However, histone H1.0 mapped on the histone H5 globular domain shows close contact of the residues at site 2 (Arg42, Arg94 and Lys97) with the linker DNA fragment and interaction of the other binding amino acid residues (Arg47, Lys69, Lys73, Arg74 and Lys85) with both dyad and linker DNA sequences, confirming the on-dyad binding mode of the histone H1.0 globular domain-DNA interaction [49]. The series of in vivo photobleaching experiments conducted on mutated histone H1.0 and H1.c [13, 49] revealed its distinct nucleosomal orientation and differential chromatin binding affinities. The nucleosome orientation of the histone H1 globular domain is determined by two conservative Lys residues, located at position 63 and 96 in histone H1c and at positions 52 and 85 in histone H1.0 [48]. However, the histone H1 binding affinity examined in the domain swap mutant containing the exchanged C-terminal domain, produced by replacing C-terminal end of H1.c with that of H1.0, was found to be reduced in the presence of mutated Lys 96 and was not affected when the residue of Lys 63 was mutated. Thus, the C-terminal domain may influence the orientation of globular domain by introducing a conformational change of the DNA [13]. In the same study, a contribution of N-terminal domain in the histone H1 binding affinity was also recognized. Due to the changes in the binding affinity which were noted between N-terminal domain swap proteins (H1c and H1.0) and wild type histone H1 variants, the N-terminal domain plays a role in the chromatin binding affinity and specificity. Presumably, the histone H1 variant-specific effect on binding affinities refers to the length of N-terminal domain and its content of Ser/Thr residues. While the histone H1.0 N-terminal domain is composed of 20 amino acid residues and contains five Ser/Thr residues, the N-terminal domain of histone H1c possessing 32 amino acid residues has only one Ser/Thr motif [13].

The distinct histone H1.0 C-terminal subdomains studied in the truncation mutants with disrupted primary sequence were found as essential for establishment of the condensed chromatin structure [5], as well as for the activation and subsequent interaction with partner protein [48]. According to these findings, the 24-amino acid residue long region is effective in mediating chromatin condensation [5], while the 47-amino acid residue long fragment is engaged in the interaction with apoptotic nuclease [50]. Thus, the amino acid composition of unique regions adopting conformations typical of disordered state, but not a primary sequence structure, determines the condensing properties of histone H1.0 [51, 52]. In fact, a detailed characteristic of histone H1.0 physicochemical parameters and its structural elements shows many features that are specific of intrinsically disordered conditions [2, 52] (Table 1).

The amino acid compositional bias, enriched in the disorder-forming amino acid residues versus low content and/or complete lack of some order-forming amino acids, as well as the negative hydropathy index and low sequence complexity, enables histone H1.0 to be recognized as a typical intrinsically disordered protein. Also, the fully disordered histone H1.0 terminal domains contain short MoRFs elements, capable of undergoing disorder-to-order transition [53]. They are usually located within the longer ANCHOR-predicted regions, representing a potential surface for recognition and binding [54]. This demonstrates that, like intrinsically disordered proteins, histone H1.0 activity is dependent on transiently formed structural conformation determining a range of temporal and reversible interactions with DNA and partner proteins. The above-mentioned studies, as well as the most recent findings (see next section) indicate that conversion from disordered (unbound) to an ordered (bound) state may be necessary for proper implementation of histone H1.0-dependent processes both in nucleus and in the nucleolus.

**Fig. 2:**
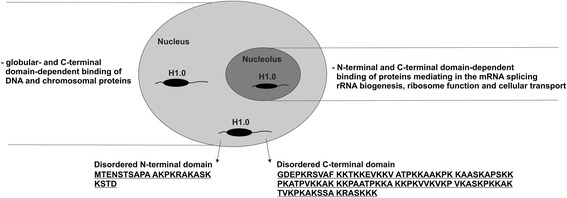
Nuclear and nucleolar functions of histone H1.0 linked to the specific amino acid composition of its disordered terminal domains. Underlined amino acids of terminal domains were predicted as disordered (PrDOS [65])

Whereas a number of earlier uncovered histone H1 variant-related interactions with nuclear and cytosolic proteins modulate DNA-dependent genomic processes (for review, see [55]), the most recent studies have confirmed histone H1.0-specific location in the nucleolus [22] and its ability to influence RNA functioning via interaction with nucleolar proteins [21] (Fig. 2).

### Histone H1.0 in nucleolar chromatin

The initial observations, originating from immunofluorescence studies with histone H1 specific antibody, indicate that a highly concentrated histone H1.0 is located close to the nucleoli in the condensed chromatin [19]. This could be indicative of the effects of histone H1.0 on the ribosomal genes activity, since its selective inhibition occurs preferentially in a dense nucleolar chromatin component [56]. However, there was no evidence of histone H1.0 presence in the nucleolus until the identification of its preferential location within the non-transcribed sequences of nucleolar-associated chromatin areas [22]. These findings are consistent with the identification of a number of nucleolar proteins as candidates for the interaction with histone H1.0 [21]. Thus histone H1.0 not only influences on the DNA activity in the late-replicating heterochromatin but may also affect the RNA metabolism in the nucleolus.

The peculiar genome regions associated with histone H1.0 were mapped by Mayor and colleagues [22]. Unlike replication-dependent histone H1.2 and a replication-independent histone H1X which are preferentially located within the LADs and primarily linked to the actively transcribed coding regions, histone H1.0 is localized mainly in the NADs, poor in AT-rich sequences and abundant in silent genes. A positive correlation between H1 histones ChIP-seq signals and the content of NAD indicates that histone H1.0 is the most enriched histone H1 variant at chromosomes that possesses a higher amount of NAD. The ChIP-seq signals also revealed enrichment of histone H1.0 in the non-transcribed rDNA sequences. Likewise, the high occupancy of histone H1.0 is characteristic for non-coding RNA genes (5S rRNA) and for the repetitive DNA sequences of acrocentric and telomeric satellites. The immunoblots of histone H1 variants abundance in cell fractions confirms the findings obtained by ChIP-seq. The histone H1.0 turned out to be mostly enriched at nucleoli, in contrast to the total cell lysate and nucleoplasm extract. Despite the confirmation of histone H1X presence in the nucleoli and a likelihood of histone H1.5 occurrence in this area, the authors state that histone H1.0 is the main nucleolar histone H1 variant in the breast cancer cells tested. Conversely, a H1.0 impoverished level was noted in HeLa cells [22] and in the MCF10-A cells, in which histone H1.0 was found to be almost undetectable [30]. However, whereas the HBP1 transcription factor induced by retinoblastoma protein (RB) enhances the activity of histone H1.0 promoter in nuclear chromatin of murine erythroleukemia cells [57], and histone H1.0 is abundantly deposited in nucleolar chromatin of breast cancer cells [22], it is intriguing whether or not histone H1.0 activity is peculiarly linked to specialized cells affecting a specific processes, i.e. related to malignant transformation, or its accumulation is widespread across various cell types and necessary for maintaining a general cell processes.

What might be helpful in answering the above question are the examination results that reveal the abundance of candidate proteins for histone H1.0 interaction. These include a group of proteins related to RNA metabolism, engaged both in the control of histone H1.0 expression [58] and in the regulation of ribosome function [21]. Among the proteins analyzed by chromatography on biotinylated H1.0 RNA and subsequently identified by mass spectrometry, the nucleolus heat shock cognate 70 kDa (Hsc70) protein was found to be co-immunoprecipitated with histone H1.0. Additionally, the majority of analyzed proteins involved in the RNA metabolism were found as mutually co-immunoprecipitated, as with anti-Hsc70 antibodies and the antibodies of RNA binding factor CSD-C2. Therefore, the histone H1.0 activity is dependent on the RNA-binding proteins, whose reciprocal interactions regulate H1.0 post-transcriptional expression [58]. Evidence for the involvement of histone H1.0 in nucleolar chromatin functioning was provided by Kalashnikova and colleagues [21], who managed to detect more than one hundred H1.0-binding proteins at the nucleolar location. In four human cell lines, the candidate histone H1.0-binding proteins, pulled-down by chimeric HaloTaq-H1.0 proteins from nuclear extracts, were detected by using LC-MS/MS. Among them, 191 protein components were detected in one replicate. In two replicates of each cell extract, 107 proteins were selected as probable histone H1.0 binding partners. Apart from 21 proteins, including histones, heat shock proteins and the proteins responsible for DNA repair, classified as miscellaneous, the rest of H1.0-binding partners were arranged into four groups according to their function. The first category encompasses 33 proteins engaged in the splicing of mRNA. They include small nuclear ribonucleoproteins, splicing factors and heterogeneous nuclear ribonucleoproteins. The second group comprises 12 proteins linked to rRNA biogenesis, as with subunit of casein kinase II and FACT complex. The third class, containing 24 proteins involved in translation, is divided into two subclasses. It includes 40S and 60S ribosomal proteins, as well as translation initiation factors. Finally, 6 elements including importins and other proteins responsible for cellular transport were incorporated into the fourth group. Whereas some of candidate H1.0-binding proteins were indirectly pulled down as the elements of protein complexes, direct H1.0-protein interaction was confirmed in the fluorescence (de) quenching experiments. This allowed splicing factors (U2AF65 and SF2/ASF) and histone chaperone FACT complex subunits (Spt16 and SSRP1) to be recognized as being capable of interacting with the histone H1.0. Also, the interaction between histone H1.0 and rarely represented proteins was confirmed by the Western blotting analyses. Protein pull down with subsequent Western blotting allowed identification of chromatin remodeler RbAp48 as interacting with histone H1.0. Moreover, whereas no interaction was identified in the presence of H1ΔCTD protein containing 97 amino acid residues corresponding to the histone H1.0 N-terminal tail and globular domain, the H1.0-RbAp48 interaction occurs in dependence of histone H1.0 C-terminal domain. A detailed analysis revealed, however, that about 25 % of the interactions between H1.0 and candidate proteins are mediated by the histone H1.0 C-terminal domain, while the remaining 75 % occur via a region encompassing the N-terminal and globular domain. The authors point out that histone H1.0 binding capability is related to its intrinsic disorder conditions, manifested mainly by a disorder-promoting amino acid composition of the C-terminal domain. The histone H1.0 C-terminal domain, enriched in charged and hydrophilic amino acids promoting disordered state [53], was also recognized as having functional subregions that promote chromatin condensation [5] and participate in protein binding [52]. Thus, disorder to order transition of the histone H1.0 C-terminal domain stretches is necessary for recognizing and binding of the partnering proteins. The same mode of operation may also correspond to the N-terminal domain, whose ability to form altered structures was confirmed in the biochemical experiment [59]. Likewise, the data originating from bioinformatics predictions [2] (Table 1 and Fig. 1) show that both N-terminal and C-terminal H1.0 domain have typical features for intrinsic disorder conditions and may serve as a protein binding surface. The changes in histone H1.0 binding properties may be determined by post-translational modifications, mainly by phosphorylation sites located both in the N-terminal and in the C-terminal domain. Phosphorylation may influence the affinity of histone H1 for DNA and affect interactions with other proteins. As a result of phosphorylation, which is dependent on the number of phosphate groups, the C-terminal domain may form differential conformations in the DNA-bound state. Phosphorylation triggers the histone H1 conformational change related to the increase in β-structure coupled to a decrease in α-helix/turns, leading to the relaxation of chromatin fiber structure [60]. Presumably, the histone H1.0 propensity for creating variously structured states determining the manifold of interactions, makes it the crucial element engaged both in the nuclear (nucleosome orientation, chromatin stabilization and transcriptional regulation) and nucleolar (rRNA synthesis and processing within the ribosome and spliceosome) chromatin [61].

Despite a lack of clear evidences concerning histone H1.0 functioning in the nucleolus, the nucleolar deposition and direct binding to a large number of multifunctional nucleolar protein partners allow histone H1.0 to be perceived as vital for the proper organization and function of the nucleolus. These findings allow us to complement the conventional histone H1 functional paradigm, i.e. chromatin compaction and transcriptional regulation realized via DNA interaction, by its chromosomal functioning through the protein-protein interactions (Fig. 2) [61].

## Conclusion

Even though the molecular and functional properties of histone H1 subtypes seem to be examined accurately, the peculiar example of histone H1.0 shows some gaps in their understanding and points to the need for their further examination. The findings published recently by Szerlog and co-workers [62] confirm histone H1 nucleolar localization and its involvement in the organization of nucleolar structure and integrity through the interaction with nucleolar proteins. The question that still remains open is whether standard and specialized histone H1 variants, which are peculiarly distributed within active and repressed genome regions [35], may contribute to the various nuclear and nucleolar processes by acting as elements of the protein-protein interaction network.

## Abbreviations

ATM, atomic-force microscopy; CHIP-seq, chromatin immunoprecipitation sequencing; DamID, DNA adenine methyltransferase identification; EZH2, enhancer of zeste 2 polycomb repressive complex 2 subunit; FACT, facilitates chromatin transcription; FRAP, fluorescence recovery after photobleaching; GAPDH, glyceraldehyde-3-phosphate dehydrogenase; LADs, lamina associated domains; LC-MS/MS, liquid chromatography-tendem mass spectrometry; MoRFs, molecular recognition features; NADs, nucleolus-associated chromatin domains; NORs, nucleolus organizer regions; PAF1, RNA polymerase II associated factor; PcG, polycomb gene complex; PRC, polycomb repressive complex

## References

[1] Happel N, Doenecke D (2009). Histone H1 and its isoforms: contribution to chromatin structure and function. Gene.

[2] Kowalski A (2015). Abundance of intrinsic structural disorder in the histone H1 subtypes. Comp Biol Chem.

[3] Hansen J, Lu X, Ross ED, Woody RW (2006). Intrinsic protein disorder, amino acid composition, and histone terminal domains. J Biol Chem.

[4] Carerino TL, Hayes JJ (2011). Structure of the H1 C-terminal domain and function in chromatin condensation. Biochem Cell Biol.

[5] Lu X, Hansen JC (2004). Identification of specific functional subdomains within the linker histone H1° C-terminal domain. J Biol Chem.

[6] Doenecke D, Alonso A (1996). Organization and expression of developmentally regulated H1° histone gene in vertebrates. Int J Dev Biol.

[7] Khochbin S, Wolffe AP (1994). Developmentally regulated expression of linker-histone variants in vertebrates. Eur J Biochem.

[8] Bouterfa HL, Triebe SM, Doenecke D (1993). Differential regulation of the human H1° histone gene transcription in human tumor cell line. Eur J Biochem.

[9] Martinez P, Vidal JM, Monsalves C, Perez M, Pucket C, Ponte I, Suau P (1995). Cloning and analysis of the coding region of the histone H1° encoding gene from rat PC12 cells. Gene.

[10] Brocard M-P, Triebe S, Peretti M, Doenecke D, Khochbin S (1997). Characterization of the two H1-encoding genes from *Xenopus laevis*. Gene.

[11] Miki BLA, Neelin JM (1975). Comparison of the histone from fish erythrocytes. Can J Biochem.

[12] Rutledge RG, Shay CE, Brown GL, Neelin JM (1981). The similarity of histone from turtle erythrocytes and liver. Can J Biochem.

[13] Vyas P, Brown DT (2012). The N- and C-terminal domains determine the differential nucleosomal binding geometry and affinity of linker histone isotypes H1^0^ and H1c. J Biol Chem.

[14] De S, Brown DT, Lu ZH, Leno GH, Wellman SE, Sittman DB (2002). Histone H1 variants differently inhibit DNA replication through an affinity for chromatin mediated by their carboxyl-terminal domain. Gene.

[15] Brown DT, Gunjan A, Alexander BT, Sittman DB (1997). Differential effect of H1 variant overproduction on gene expression is due to differences in the central globular domain. Nucleic Acids Res.

[16] Brown DT, Izard T, Misteli T (2006). Mapping the interaction surface of linker histone H1.0 with the nucleosome of native chromatin in vivo. Nat Struct Mol Biol.

[17] Uversky VN (2013). A decade and a half of protein intrinsic disorder: biology still waits for physics. Protein Sci.

[18] Roque A, Ponte I, Suau P. Interplay between histone H1 structure and function. Biochim Biophys Acta. 2015. doi:10.1016/j.bbagrm.2015.09.009. 10.1016/j.bbagrm.2015.09.009 26415976

[19] Breneman JW, Yau P, Teplitz RL, Bradbury EM (1993). A light microscope study of linker histone distribution in rat metaphase chromosomes and interphase nuclei. Exp Cell Res.

[20] Gorka C, Fakan S, Lawrence JJ (1993). Light and electron microscope immunocytochemical analyses of histone H1° distribution in the nucleus of Friend erythroleukemia cells. Exp Cell Res.

[21] Kalashnikova AA, Winkler DD, McBryant SJ, Henderson RK, Herman JA, DeLuca JG, Luger K, Prenni JE, Hansen JC (2013). Linker histone H1.0 interacts with an extensive network of proteins found in the nucleolus. Nucleic Acids Res.

[22] Mayor R, Izquierdo-Bouldstridge A, Millan-Arino L, Bustillos A, Sampaio C, Luque N, Jordan A (2015). Genome distribution of replication-independent histone H1 variants shows H1.0 associated with nucleolar domains and H1X with RNA polymerase II-enriched regions. J Biol Chem.

[23] Routh A, Sandin S, Rhodes D (2008). Nucleosome repeat length and linker histone stoichiometry determine chromatin fiber structure. Proc Natl Acad Sci U S A.

[24] Kowalski A, Pałyga J (2012). Linker histone subtypes and their allelic variants. Cell Biol Int.

[25] Millãn-Ariño L, Izquierdo-Bouldstridge A, Jordan A. Specificities and genomic distribution of somatic mammalian histone H1 subtypes. Biochim Biophys Acta. 2015. doi:10.1016/j.bbagrm.2015.10.013. 10.1016/j.bbagrm.2015.10.013 26477490

[26] Sirotkin AM, Edelmann W, Cheng G, Klein-Szanto A, Kucherlapati R, Skoultchi AI (1995). Mice develop normally without the H1° linker histone. Proc Natl Acad Sci U S A.

[27] Fun Y, Sirotkin AM, Russel RG, Ayala J, Skoultchi AI (2001). Individual somatic H1 subtypes are dispensable for mouse development even in mice lacking the H1(0) replacement subtype. Mol Cell Biol.

[28] Fun Y, Nikitina T, Morin-Kensicki EM, Zhao J, Magnuson TR, Woodcock CL, Skoultchi AI (2003). H1 linker histones are essential for mouse development and affect nucleosome spacing in vivo. Mol Cell Biol.

[29] Sancho M, Diani E, Beato M, Jordan A (2008). Depletion of human histone H1 variants uncovers specific roles in gene expression and cell growth. PLoS Genet.

[30] Herghet SP, Schneider R (2015). The H1 linker histones: multifunctional proteins beyond the nucleosomal core particle. EMBO Rep.

[31] Crane-Robinson C. Linker histones: history and current perspectives. Biochim Biophys Acta. 2015. doi:10.1016/j.bbagrm.2015.10.008. 10.1016/j.bbagrm.2015.10.008 26459501

[32] Parseghian MH (2015). What is the role of histone H1 heterogeneity ?. AIMS Biophys.

[33] Th’ng JP, Sung R, Ye M, Hendzel MJ (2005). H1 family histone in the nucleus. Control of binding and localization by the C-terminal domain. J Biol Chem.

[34] Clausell J, Happel N, Hale TK, Doenecke D, Beato M (2009). Histone H1 subtypes differentially modulate chromatin condensation without preventing ATP-dependent remodeling by SWI/SNF or NURF. PLoS One.

[35] Izzo A, Kamieniarz-Gdula K, Ramirez F, Noureen N, Kind J, Manke T, van Steensel B, Schneider R (2013). The genomic landscape of the somatic linker histone subtypes H1.1 to H1.5 in human cells. Cell Rep.

[36] Sarg B, Lopez B, Lindner H, Ponte I, Suau P, Roque A (2014). Sequence conservation of linker histones between chicken and mammalian species. Data Brief.

[37] Millãn-Ariño L, Islam ABMMK, Izquierdo-Bouldstridge A, Mayor R, Therme J-M, Luque N, Sancho M, Lopez-Bigas N, Jordan A (2014). Mapping of six somatic histone H1 somatic variants in human breast cancer cells uncovers specific features of H1.2. Nucleic Acids Res.

[38] Kim K, Lee B, Kim J, Choi J, Kim JM, Xiong Y, Roeder NG, An W (2013). Linker histone H1.2 cooperates with Cul4A and PAF1 to drive H4K31 ubiquitylation-mediated transactivation. Cell Rep.

[39] Kim JM, Kim K, Punj V, Liang G, Ulmer TS, Lu W, An W. Linker histone H1.2 establishes chromatin compaction and gene silencing through recognition of H3K27me3. Sci Rep. 2015;5. doi:10.1038/srep16714. 10.1038/srep16714 PMC4652225 26581166

[40] Panyim S, Chalkley R (1969). A new histone found only in mammalian tissues with little cell division. Biochem Biophys Res Commun.

[41] Gjerset R, Gorka C, Hasthorpe S, Lawrence JJ, Eisen H (1982). Developmental and hormonal regulation of histone H1^0^ in rodents. Proc Natl Acad Sci U S A.

[42] Rousseau D, Khochbin S, Gorka C, Lawrence JJ (1991). Regulation of histone H1° accumulation during induced differentiation of murine erythroleukemia cell. J Mol Biol.

[43] Roche J, Gorka C, Goeltz P, Lawrence JJ (1985). Association of histone H1(0) with a gene repressed during liver development. Nature.

[44] Delabar JM (1985). Nonrandom location of H1-H1° histones on chromatin of mouse liver and brain. J Biol Chem.

[45] Brown DT, Alexander BT, Sittman DB (1996). Differential effect of H1 variant overexpression on cell cycle progression and gene expression. Nucleic Acids Res.

[46] Bhan S, May W, Warren SL, Sittman DB (2008). Global gene expression analysis reveals specific and redundant roles for H1 variants, H1c and H1(0), in gene expression regulation. Gene.

[47] Therme J-M, Sesé B, Millán-Ariño L, Mayor R, Izpisúa Belmonte JC, Barrero MJ, Jordan A (2011). Histone H1 variants are differentially expressed and incorporated into chromatin during differentiation and reprogramming to pluripotency. J Biol Chem.

[48] George EM, Izard T, Anderson SD, Brown DT (2010). Nucleosome interaction surface of linker histone H1c is distinct from that of H1^0^. J Biol Chem.

[49] Zhou B-R, Jiang J, Feng H, Ghirlando R, Sam Xiao T, Bai Y (2015). Structural mechanism od nucleosome recognition by linker histones. Mol Cell.

[50] Widłak P, Kalinowska M, Parseghian MH, Lu X, Hansen JC, Garrard WT (2005). The histone H1 C-terminal domain binds to the apoptotic nuclease DNA fragmentation factor (DFF40/CAD) and stimulates DNA cleavage. Biochemistry.

[51] Lu X, Hamkalo B, Parseghian MH, Hansen JC (2009). Chromatin condensing functions of the linker histone C-terminal domain are mediated by specific amino acid composition and intrinsic protein disorder. Biochemistry.

[52] McBryant S, Hansen JC. Dynamic fuzziness during linker histone action. In: Fuxreiter M, Tompa P, editors. Fuzziness: Structural Disorder in Protein Complexes. New York: Landes Bioscience; Austin: Springer Science + Business Media; 2012. p. 15–22.

[53] Mohan A, Oldfield CJ, Radivojac P, Vacic V, Cortese MS, Dunker AK, Uversky VN (2006). Analysis of molecular recognition features (MoRFs). J Mol Biol.

[54] Dosztànyi Z, Mészàrosz B, Simon I (2009). ANCHOR: web server for predicting protein binding regions in disordered proteins. Bioinformatics.

[55] McBryant SJ, Lu X, Hansen JC (2010). Multifunctionality of the linker histones: an emerging role for protwein-protein interactions. Cell Res.

[56] Thiry M, Schee U, Goessens G (1991). Localization of nucleolar chromatin by immunocytochemistry and in situ hybridization at the electron microscopic level. Electron Microsc Rev.

[57] Lemercier C, Duncliffe K, Boibessot I, Zhang H, Verdel A, Angelov D, Khochbin S (2000). Involvement of retinoblastoma protein and HPB1 in histone H1^o^ gene expression. Mol Cell Biol.

[58] Di Liegro CM, Schiera G, Proia P, Saladino P, Di Liegro I (2013). Identification in the rat brain of a set of nuclear proteins interacting with H1° mRNA. Neurosience.

[59] Vila R, Ponte I, Collado M, Arondo JL, Jimenez MN, Rico M, Suau P (2001). DNA induced α-helical structure in the NH_2_-terminal domain of histone H1. J Biol Chem.

[60] Lopez R, Sarg B, Lindner H, Bartolome S, Ponte I, Suau P, Roque A. Linker histone partial phosphorylation: effects on secondary structure and hromatin condensation. Nucleic Acids Res. 2015;1. doi:10.1093/nar/gkv304. 10.1093/nar/gkv304 PMC4482070 25870416

[61] Kalashnikova AA, Rogge RA, Hansen JJ. Linker histone H1 and protein-protein interactions. Biochim Biophys Acta. 2015. doi:10.1016/j.bbagrm.2015.10.004. 10.1016/j.bbagrm.2015.10.004 PMC4775371 26455956

[62] Sherlog HJ, Herman JA, Krause CM, DeLuca JG, Skoultchi A, Winger QA, Prenni JE, Hansen JC (2015). Proteomic characterization of the nucleolar linker histone H1 interaction network. J Mol Biol.

[63] Vacic V, Uversky VN, Dunker AK, Lonardi S. Composition profiler: a tool for discovery and visualization of amino acid composition differences. BMC Bioinformatics. 2007. doi:10.1186/1471-2105-8-211. 10.1186/1471-2105-8-211 PMC1914087 17578581

[64] Ishida T, Kinoshita K (2007). PrDos: prediction of disordered protein regions from amino acid sequence. Nucleic Acids Res.

[65] Peng Z, Mizianty MJ, Kurgan L (2014). Genome-scale prediction of proteins with long intrinsically disordered regions. Proteins.

[66] Disfani FM, Hsu W-L, Mizianty MJ, Oldfielf CJ, Xue B, Dunker AK, Uversky VN, Kurgan L (2012). MoRFpred, a computational tool for sequence-based prediction and characterization of short disorder-to-order transitioning binding regions in proteins. Bioinformatics.

[67] Wootton JC (1994). Non-globular domains in protein sequences: automatic segmentation using complexity measures. Comput Chem.

